# Insect prophenoloxidase: the view beyond immunity

**DOI:** 10.3389/fphys.2014.00252

**Published:** 2014-07-11

**Authors:** Anrui Lu, Qiaoli Zhang, Jie Zhang, Bing Yang, Kai Wu, Wei Xie, Yun-Xia Luan, Erjun Ling

**Affiliations:** Key Laboratory of Insect Developmental and Evolutionary Biology, Institute of Plant Physiology and Ecology, Shanghai Institutes for Biological Sciences, Chinese Academy of SciencesShanghai, China

**Keywords:** insect, prophenoloxidase, type-3 copper proteins, protein structure, melanization

## Abstract

Insect prophenoloxidase (PPO) is an important innate immunity protein due to its involvement in cellular and humoral defense. It belongs to a group of type-3 copper-containing proteins that occurs in almost all organisms. Insect PPO has been studied for over a century, and the PPO activation cascade is becoming clearer. The insect PPO activation pathway incorporates several important proteins, including pattern-recognition receptors (PGRP, β GRP, and C-type lectins), serine proteases, and serine protease inhibitors (serpins). Due to their complexity, PPO activation mechanisms vary among insect species. Activated phenoloxidase (PO) oxidizes phenolic molecules to produce melanin around invading pathogens and wounds. The crystal structure of *Manduca sexta* PPO shows that a conserved amino acid, phenylalanine (F), can block the active site pocket. During activation, this blocker must be dislodged or even cleaved at the N-terminal sequence to expose the active site pockets and allow substrates to enter. Thanks to the crystal structure of *M. sexta* PPO, some domains and specific amino acids that affect PPO activities have been identified. Further studies of the relationship between PPO structure and enzyme activities will provide an opportunity to examine other type-3 copper proteins, and trace when and why their various physiological functions evolved. Recent researches show that insect PPO has a relationship with neuron activity, longevity, feces melanization (phytophagous insects) and development, which suggests that it is time for us to look back on insect PPO beyond the view of immunity in this review.

## Introduction

Type-3 copper proteins have two copper ions and three histidines (H) in each active site pocket (Ashida and Brey, 1997; Aguilera et al., 2013). This group of proteins is distributed extensively among almost all organisms, including vertebrates, invertebrates, plants, and microbes (Aguilera et al., 2013). Type-3 copper proteins from different organisms are named differently; e.g., tyrosinase in mammals and microbes, prophenoloxidase (PPO) in insects and crabs, polyphenol oxidase (also termed as PPO) in plants, and hemocyanin in arthropods (Cerenius et al., 2008; Aguilera et al., 2013). Each type-3 copper protein has a different physiological function. Mammalian tyrosinase activity is closely related to skin and hair color, and loss of tyrosinase activity in humans is the direct cause of albinism and leucoderma (Oetting and King, 1999; Kirkwood, 2009). Activation of plant polyphenol oxidase induces the browning of food, decreasing its qualities (Aquino-Bolaños and Mercado-Silva, 2004). However, polyphenol oxidase can also affect the production of tea volatiles (Harbowy and Balentine, 1997). Arthropod hemocyanin mainly transfers oxygen in the hemolymph (van Holde and Miller, 1995), while tyrosinase in microbes is positively related to pathogenicity (Mayer, 2006; Shang et al., 2012) and, in insects and other arthropods, PPO is an important innate immunity protein (Ashida and Brey, 1997; Cerenius et al., 2008; Kanost and Gorman, 2008). Insect hemolymph melanization induced by the PPO was first recorded in 1898 (Biedermann and Moritz, 1898). Over the last three to four decades, many laboratories have worked cooperatively on the PPO activation pathway and its regulation (Ashida and Brey, 1997; Cerenius et al., 2008; Kanost and Gorman, 2008), and it is now clear that PPO activation occurs through a cascade of patterns-recognition proteins, serine proteases, and serine protease inhibitors (serpin) after initial pathogen detection (Ashida and Brey, 1997; Cerenius et al., 2008; Kanost and Gorman, 2008). In some insects, serine protease homologs (SPH) are involved in PPO activation (Ross et al., 2003; Yu et al., 2003). In addition, insect PPO is also responsible for wound healing and hemolymph clotting (Lai et al., 2002; Ramet et al., 2002; Galko and Krasnow, 2004; Karlsson et al., 2004). However, the recent knock-down and knock-out of PPO in *Tribolium castaneum* and *Drosophila melanogaster* indicate that insect PPO does not determine cuticle sclerotization (Shao et al., 2012; Binggeli et al., 2014).

In insects, the innate immune system is composed mainly of cellular and humoral immunity (Strand, 2008). Cellular immunity includes phagocytosis of small invading microbes and the encapsulation of large parasites by circulating hemocytes (Lavine and Strand, 2002; Strand, 2008). Humoral immunity is induced by humoral antibacterial peptides (AMP) produced via the Toll and/or immune deficiency (Imd) pathways, as well as many other immunity proteins (Lemaitre and Hoffmann, 2007). PPO is a humoral protein that can induce melanization around invading pathogens after activation, and induces cellular and humoral immunity simultaneously (Lemaitre and Hoffmann, 2007). Intermediates produced in the melanization process can kill bacteria directly (Zhao et al., 2007). When PPO was knocked down via RNAi, invertebrate animals were easily infected by pathogenic bacteria and viruses (Liu et al., 2007; Paria et al., 2013). After knock-down of PPO in *Aeromonas hydrophila*, phagocytosis and nodule formation were reduced and, eventually, bacteria in the hemolymph multiplied and caused mortality (Liu et al., 2007). When *D. melanogaster* PPO1 and PPO2 (DmPPO1 and DmPPO2) were deleted, the mutants (PPO1^Δ^, PPO2^Δ^) were more susceptible to infection by gram-positive bacteria and fungi (Binggeli et al., 2014). Thus, PPO is an important immunity protein in both insects and other invertebrates, as has been reviewed in several studies (Ashida and Brey, 1997; Cerenius et al., 2008; Kanost and Gorman, 2008; González-Santoyo and Córdoba-Aguilar, 2012). In this review, we focus on information beyond the involvement of PPO in immune responses, mainly in insects and some other invertebrates.

## Distribution of PPO

In insects, circulating hemocytes has been viewed as the only source of PPO (Ashida and Brey, 1997). In the silkworm *Bombyx mori* (*B. mori*) and other Lepidoptera, oenocytoids produce PPO (Strand, 2008; Liu et al., 2013), whereas in *D. melanogaster*, crystal cells produce PPO (Rizki et al., 1985). However, other types of hemocyte may have PPO in insects and other invertebrates; for example, in *Carcinus maenas*, granulocytes have PPO (Söderhäll and Smith, 1983). Recent work indicates that some prohemocytes, granulocytes and plasmatocytes also have PPO in *B. mori* (Ling et al., 2005), and PPO-positive hemocytes have been identified in *B. mori* hematopoietic organs (Wang et al., 2010). Staining reveals that some granulocytes and spherulocytes in *Manduca sexta* also have PPO (Ling and Yu, 2005). Moreover, immunostaining of living hemocytes using antibodies against *M. sexta* PPO revealed that PPO binds to the membranes of granulocytes and spherulocytes, but not to those of oenocytoids (Ling and Yu, 2005). In *Culex pipiens quinquefasciatus*, oenocytoids, prohemocytes, and granulocytes may be PPO-positive, but depend on the developmental stage and even the extent of blood feeding (Wang et al., 2011). Thus, the distribution of PPO protein in hemocytes is not limited to one type. Since it wasn't accomplished so far to co-localize the levels of *in situ* transcription and PO activity (both activation and staining) on the same cells because they require different assays, it is difficult to conclusively show that PPO-positive hemocytes can produce PPO. However, plasma PPO contamination can be ruled out because anticoagulant buffers have been used in some studies. Furthermore, not all hemocytes were positively stained, based on simultaneous observations using a microscope.

A recent study showed that epidermal cells in the hindgut of *B*. *mori* also produce PPO (Shao et al., 2012); the authors used various techniques to show that the cells contained signals associated with PPO proteins, transcription, and activity. To prevent contamination, circulating hemocytes were pre-labeled via phagocytosis of injected fluorescent beads. However, no fluorescent beads were found in the hindgut, which indicates that any PPO-positive cells present in the midgut were not hemocytes. The authors also used lysozyme, an immunity protein produced in response to an immune challenge, as a probe to show that there is no direct physical exchange between the hindgut and plasma. Therefore, PPO in the hindgut was from neither hemocytes nor plasma contamination. The wild-type *D. melanogaster* larval hindgut was also positively stained (Shao et al., 2012), as described above. However, when both DmPPO1 and DmPPO2 (PPO1^Δ^, PPO2^Δ^) were deleted (Binggeli et al., 2014), the larval hindguts of those mutants were not positively stained (Lu, personal observations). All species of insects assayed in the study had PPO in their hindguts according to the staining results (Shao et al., 2012).

PPO was also found in other tissues. For example, wing discs dissected from *B. mori* larvae can release PPO into the culture medium. However, PPO-positive cells in wing disc cavities might derive from the attached hematopoietic organ (Diao et al., 2012). Since wing discs are physically connected with the hematopoietic organ via many small tubes (Ling et al., 2006), some hemocytes may be accidentally released into the wing discs when PPO is released. PPO has also been identified in the hind wing of *T. castaneum* by MALDI/TOF (Dittmer et al., 2012). Insect cuticle has PPO (Ashida and Brey, 1997). No PPO mRNA signals were detected in the silkworm epidermal cells, and the authors concluded that cuticular PPO was transferred from the hemolymph (Asano and Ashida, 2001). Biochemical assays indicate that cuticular PPO is modified during its transfer from hemolymph to cuticle (Asano and Ashida, 2001). According to proteomics studies, PPO also occurs in some other tissues, including the silk gland, trachea, and adult scales (Fu et al., 2011; Dong et al., 2013).

PPO has no signal peptides (Ashida and Brey, 1997; Cerenius et al., 2008; Kanost and Gorman, 2008). Thus, it is thought to be released from hemocytes after lysis (Ashida and Brey, 1997), which is regulated by the JNK pathway in *D. melanogaster* (Bidla et al., 2007). Eicosanoids can also regulate PPO releasing from oenocytoids (Shrestha and Kim, 2008). However, the mechanism underlying PPO is releasing from hindgut cells is at present unclear; it may be regulated by an unknown mechanism.

## PPO detection

Immunostaining and *in situ* hybridization is the best methods of determining protein or transcript levels when searching for cellular proteins. For example, immunostaining and *in situ* hybridization of *B. mori* and *M. sexta* showed that their oenocytoids produce PPO (Iwama and Ashida, 1986; Jiang et al., 1997). Antibodies that can detect PPO in one insect species may not cross-identify PPO in others. However, there are several simple methods for detecting insect PPO in cells and tissues. Insect PPO can be activated by many cationic or anionic detergents and alcohols (e.g., methanol, ethanol, and 2-propanol) via unknown mechanisms (Ashida and Brey, 1997). Ethanol can activate PPO even within cells and tissues, and PPO-positive cells appear if the substrates are subsequently replaced (Ling et al., 2005). Using this method, new types of PPO-positive hemocytes and tissues have been identified without using the immunostaining method (Ling and Yu, 2005; Ling et al., 2005; Diao et al., 2012; Shao et al., 2012). This method can also be used to detect PPO in native gels; for example, DmPPO1 and DmPPO2 appear as different bands on the same native gel (Asano and Takebuchi, 2009). A native gel assay showed that PPO was released separately from cultured larval wing discs and hindguts (Diao et al., 2012; Shao et al., 2012). After deletion of one of the two disulfides in DmPPO1, the enzyme activities of mutants decreased significantly on the same native gel (Lu et al., 2014b). In other studies, DmPPO1 was over-expressed in S2 cells for 48 h with and without Cu^2+^ added (Chen et al., 2012; Liu et al., 2012). When Cu^2+^ was absent, PPO did not appear unless the native gel was pre-incubated in a buffer containing Cu^2+^ and, compared to the treatment that included Cu^2+^, the two bands were shifted (Liu et al., 2012). This indicates that PPO expression and Cu^2+^ chelation into the active site pockets are independent processes. Two Cu^2+^ ions can significantly alter the electric charge of PPO, but this unique property would not have been found without resolution of the corresponding PPOs on a native gel. Besides PPO, enzymes such as laccase and peroxidase also oxidize some phenols to produce melanin (Kanost and Gorman, 2008). Therefore, it is necessary to identify such enzymes when a staining method based on enzyme activity is used. PPO is the only enzyme activated by ethanol (Shao et al., 2012). When their specific substrates and strong inhibitors are taken into considerations, identifying the enzyme responsible for the staining is relatively easy (Shao et al., 2012). Therefore, PPO can be quickly detected by taking advantage of its activation by ethanol and other detergents.

## Expression of recombinant PPO in eukaryotic and prokaryotic cells

Purification of native insect PPO is challenging since it is easily activated during preparations. However, studying its biochemical properties requires sufficient purified PPO. It may be helpful to produce recombinant PPO in this context. *Spodoptera litura* PPO (SlPPO) was also expressed in *E. coli* at 37°C (Rajagopal et al., 2005), but there was no solid evidence that the recombinant SlPPO had PO activity. In *D. melanogaster*, there are three PPO genes: DmPPO1 (CG5779), DmPPO2 (CG8193), and DmPPO3 (CG2952). Recently, three PPOs from *D. melanogaster* (DmPPO) were over-expressed in S2 cells, and all had PO activities if additional Cu^2+^ was added to the culture medium (Liu et al., 2012). By contrast, only DmPPO1 and DmPPO2 were expressed in wild-type *D. melanogaster* (Tang, 2009; Binggeli et al., 2014). Another study found that recombinant DmPPOs were expressed in *E. coli* at 16°C, yielding soluble DmPPO (Li et al., 2012). These recombinant DmPPO proteins showed PO activity upon activation by cetylpyridinium chloride (CPC), ethanol, and serine protease. In an *E. coli* expression system, Cu^2+^ was not necessary during protein expression (Li et al., 2012), and its absence yielded apo-PPO and avoided PPO activation during purification. Conversely, Cu^2+^ can be added to transform apo-PPO into holo-PPO for later PO activity assays. The expression of insect PPO in *E. coli* is helpful for understanding the relationship between its structure and enzyme activity (Chen et al., 2012; Lu et al., 2014b), and its activation *in vitro* (Lu et al., 2014a). The mosquito genome contains up to 10 PPO genes (Christophides et al., 2002; Waterhouse et al., 2007), and we expect that, in the future, these PPOs will be separated over-expressed to facilitate evaluations of their biochemical properties, and anti-bacterial and -malarial activities.

## PPO structure and activities

Determination of the crystal structure of a protein is important for understanding its functions. The crystal structures of several type-3 copper proteins, including hemocyanins, catechol oxidase, and bacterial tyrosinase, have been determined (Gerdemann et al., 2002; Kusche et al., 2003; Sendovski et al., 2011), and that of MsPPO has also been determined using purified native *M. sexta* PPO (Li et al., 2009). The latter crystal structure shows that *M. sexta* PPO is a heterodimer with MsPPO1 and MsPPO2 formed back-to-back (Li et al., 2009). MsPPO has two copper atoms (CuA and CuB) in the active site pocket and two disulfide bonds in each monomer, as predicted. A conserved Phe (F) residue called as the place holder occurs in the active site pocket before activation and is thought to block access to the substrate. When PPO is activated, this place holder must be removed from the active site pocket. When the blocking residue (F^84^) in DmPPO3 was mutated into tryptophan (W), which has a hydrophobic side chain, DmPPO3(W^84^) activity significantly decreased after being activated by ethanol (Chen et al., 2012).

DmPPO3 is unique to *D. melanogaster*. When *D. melanogaster* was infected by parasites, lamellocytes rapidly differentiated (Labrosse et al., 2005). However, in mutant hop^Tum−l^ with enriched lamellocytes, PPO3 was expressed with accompanying spontaneous melanization (Luo et al., 1995; Nam et al., 2008). When DmPPO3 was overexpressed in various *D. melanogaster* tissues using a UAS-GAL4 system, the corresponding tissues were melanized (Nam et al., 2008), indicating that PPO3 can auto-activate independently of serine protease cleavage (Liu et al., 2012). The addition of Cu^2+^ or substrate L-DOPA to culture media resulted in auto-melanization of DmPPO3-expressing S2 cells, which was not due to serine protease cleavage (Nam et al., 2008; Liu et al., 2012).

Three *D. melanogaster* PPOs were modeled from the predicted MsPPO crystal structures (Chen et al., 2012), and the blocking residue was removed to expose the active site pocket of each PPO using Pymol. Compared to DmPPO1 and DmPPO2, DmPPO3 has a large active pocket, and thus some substrate may enter through the gap between the place holder and entrance. The model predicted that two important amino acids (I^218^ in Copper A and A^393^ in Copper B of DmPPO3) would affect the size of active site pocket. When these two amino acids were mutated to reduce the size of the active site pocket, the auto-activation property of DmPPO3 was restored (Chen et al., 2012). The corresponding amino acids in other insect PPOs are also variable (Chen et al., 2012).

DmPPO1 and DmPPO2 have a loop extending out of the protein at the C-terminus (Chen et al., 2012), but DmPPO3 has lost this fragment and has a short helix in its place. When the corresponding sequence from DmPPO1 was added to DmPPO3, the auto-activation of mutant DmPPO3 decreased significantly. Therefore, the missing fragment may also influence DmPPO3 auto-activation. Deletion of the corresponding sequence affects the activity of DmPPO2 but not of DmPPO1 after a activation by ethanol. Notably, the sequence corresponding to the extending loop lost a variable number of amino acids in most mosquito PPOs (Chen et al., 2012). Elucidating the sequences of amino acids that can affect DmPPO3 activity is challenging if we have no MsPPO crystal structure for reference.

In *M. sexta*, the tight heterodimer structure of MsPPO1 and MsPPO2 is formed through extensive hydrophobic and charge-charge interaction (Li et al., 2009). This suggests that it may be possible to tag another protein at the C-terminus of insect PPO. The fused protein DmPPO-GFP was expressed in S2 cells and *E. coli* with green fluorescence and PO activity, and they were also activated by ethanol and serine protease separately (Yang et al., 2013). In future, *in vivo* expression of PPO-GFP would facilitate investigation of its functions in cellular and humoral immunity.

Most insect PPOs and arthropod hemocyanin have two disulfide bonds at the C-terminus. However, each PPO in the parasitic wasp *Pimpla hypochondriaca* has only one disulfide bond (Parkinson et al., 2001). Protein folding and stability can be improved by disulfide bond formation (Sevier and Kaiser, 2002). Deletion of one or both disulfide bonds in DmPPO1 did not cause DmPPO1 to break down (Lu et al., 2014b), but the activities of DmPPO1 expressed in S2 cells and *E. coli* decreased significantly. Deletion of disulfide bonds also reduced thermostability of DmPPO1. Furthermore, the antibacterial activity of DmPPO1 in which one or both of the disulfide bonds had been deleted was decreased (Lu et al., 2014b). Since type-3 copper proteins exist in almost all organisms (Aguilera et al., 2013), this group of proteins may be suitable markers for the study of evolution. The identification of important amino acids and fragments in insect PPO and other type-3 copper proteins may aid the understanding of when, why, and how type-3 copper proteins adapt to different environments and evolve to serve different physiological functions.

## PPO activation

**Figure 1:**
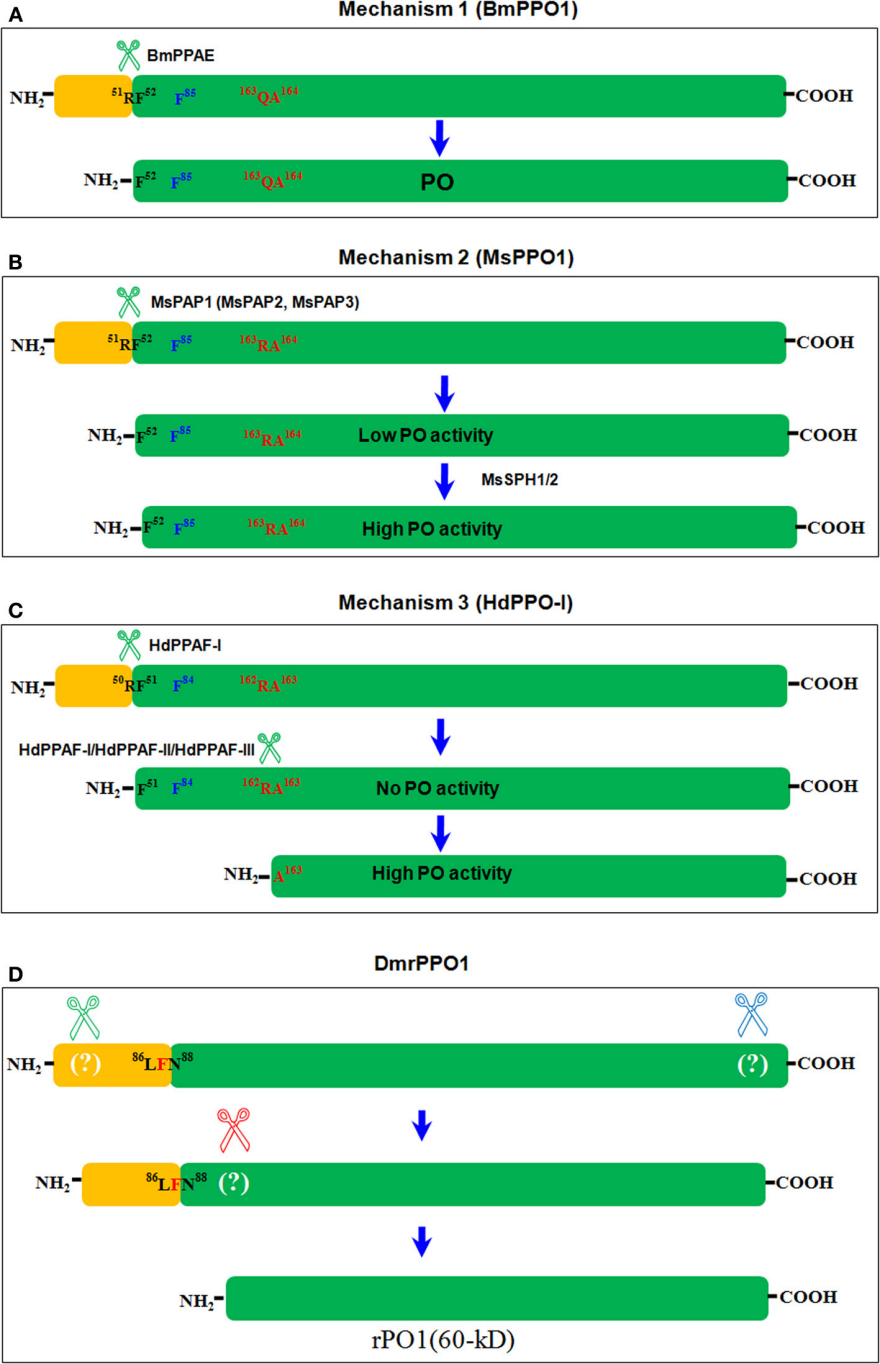
**Three mechanisms of insect PPO activation**. The terminal serine protease cleaves PPO differently among insect species (Ashida and Brey, 1997; Lee et al., 1998; Kim et al., 2002; Kanost and Gorman, 2008). And the three mechanisms diverge based on these differences. Here, we consider only BmPPO1 (Accession: NP_001037335), MsPPO1 (Accession: O44249), and HdPPO1 (Accession: BAC15603) as a summary. The conserved bonds ^51^RF^52^ in BmPPO1 **(A)**, ^51^RF^52^ in MsPPO1 **(B)**, and ^50^RF^51^ in HdPPO1 **(C)** were cleaved by serine proteases separately in the same way, as shown. F^85^ in BmPPO1, F^85^ in MsPPO1, and F^84^ HdPPO1 (labeled in blue) function as the place holder in the respective active site pockets, and may block substrates until they are dislodged. In HdPPO1, ^162^RA^163^ was further cleaved to form a fragment at 60 kD with PO activity **(C)**. The corresponding sequences in BmPPO1 **(A)** and MsPPO1 **(B)** are also shown in red. In *B. mori*, BmPPO was cleaved by PPAE to produce PO (Ashida and Brey, 1997) **(A)**. In *M. sexta*, PAPs cleaved MsPPO in the same place as in *B. mori* to produce PO fragments with low enzyme activity. When SPHs were added, PO activity increased significantly (Kanost and Gorman, 2008) **(B)**. In *H. diomphalia*, HdPPO was cleaved as in BmPPO and MsPPO by PPAF-I at the conserved bond ^50^RF^51^. However, the large fragment had no PO activity unless PPAF-I, PPAF-II, and PPAF-III were combined, and was cleaved again at ^162^RA^163^ to produce a fragment at 60 kD; this fragment had PO activity (Lee et al., 1998; Kim et al., 2002) **(C)**. In an *in vitro* assay, commercial α-chymotrypsin cleaved *D. melanogaster* recombinant PPO1 (Accession: AAF57775) (rPPO1) in at least three places to produce a fragment, also of ~60 kD, with direct enzyme activity (Lu et al., 2014a) **(D)**.

Melanization catalyzed by active phenoloxidase (PO) (PO is cleaved into PO by serine proteases) in insects is central to sequestering invading pathogens and healing wounds (Ashida and Brey, 1997; Lai et al., 2002; Kanost and Gorman, 2008; Nam et al., 2012). In insect hemolymph, PO exists as PPO zymogen that must be activated by proteolytic cleavage (Ashida and Brey, 1997; Cerenius et al., 2008; Kanost and Gorman, 2008). A serine proteinase cascade for insect PPO activation was proposed in 1986 (Yoshida and Ashida, 1986). Subsequently, many proteins—such as PPO-activating enzyme (PPAE) (Ashida and Brey, 1995, 1997), PPO-activating proteinases (PAPs) (Jiang et al., 2003a,b, 2011), *D. melanogaster* protease MP1, MP2, and Hayan (Tang, 2009; Nam et al., 2012; An et al., 2013), serine proteinase homologs (SPHs) (Ross et al., 2003; Yu et al., 2003), PPO-activating factor (PPAF) (Lee et al., 1998), serpins (Michel et al., 2005; Bruning et al., 2007; Scherfer et al., 2008; Jiang et al., 2009; Zou et al., 2010), C-type lectins (Yu et al., 1999), β-1,3-glucan recognition proteins (β GRP) (Yu et al., 2002; Lee et al., 2004), Gram-negative bacteria-binding proteins (GNBP), and peptidoglycan recognition proteins (PGRP) (Takehana et al., 2002)—have been found to positively or negatively regulate PPO activation using *B. mori*, *M. sexta, D. melanogaster, Holotrichia diomphalia*, and mosquitoes as models. A detailed pathway for PPO activation in *M. sexta* was put forward following a number of biochemical studies (Jiang et al., 2003a,b; Yu et al., 2003; Zou and Jiang, 2005; Kanost and Gorman, 2008). Briefly, in response to microorganism infection, hemolymph pattern-recognition proteins (PGRP, ß GRP, and C-type lectin) bind to the microorganism's surface polysaccharides, inducing initiator protease(s) activation. Next, the initiator protease triggers a protease cascade, activating terminal serine proteases such as PPAE, PAP, or PPAF to cleave PPO and form active PO (Cerenius et al., 2008; Kanost and Gorman, 2008). Other proteases are likely involved in the process. For example, various serpins can limit the activity of corresponding proteinases, thereby limiting the reaction speed and avoiding excessive melanization *in vivo* (Kanost, 1999; Kanost et al., 2004). However, the literatures indicate that there are differences in the final step of PPO cleavage to produce activated PO in different species of insects. Three mechanisms are summarized as below (Figure 1).

### Mechanism 1

In *B. mori*, PPAE purified from cuticles directly cleaved BmPPO into active PO (Ashida and Brey, 1997). The N-terminus amino acid sequences of POs showed that *B. mori* PPO1 (78.78 kD) and PPO2 (80.12 kD) were both cleaved at ^51^RF^52^, and large fragments (BmPO1, 72.82 kD; BmPO2, 74.25 kD) had direct PO activity (Figure 1A) (Yasuhara et al., 1995; Ashida and Brey, 1997). In the BmPPO activation cascade, PGRP and ß GRP detect invading microorganisms, and PPAE is subsequently cleaved and activated (Ashida et al., 1983; Ashida and Brey, 1997). BAEE and Ca^2+^ are involved in this pathway, but BAEE does not directly activate PPAE (Yoshida and Ashida, 1986; Ashida and Brey, 1997). Recently, several proteins that function in silkworm nodule melanization have been identified (Sakamoto et al., 2011; Chen et al., 2014; Tokura et al., 2014), most of which are similar to the MsPPO activation pathway. The PPO activation pathway in *B. mori* will likely be clarified in the future.

### Mechanism 2

In *M. sexta*, MsPPO is activated via a more complicated mechanism than in *B. mori*, and biochemical studies have identified many proteins that regulate PPO activation (Jiang et al., 2003a,b; Yu et al., 2003; Zou and Jiang, 2005; Zou et al., 2005). PPO activation in *M. sexta* is also initiated when the corresponding pattern-recognition receptors bind to elicitors on invading microorganisms, which is similar in most insects. Three PAPs are known to cleave PPO directly at the same conserved sequence (^51^RF^52^ in MsPPO1; ^49^RV^50^ in MsPPO2) where PPAE cleaved BmPPO (Ashida and Brey, 1997; Jiang et al., 2003a,b). However, the large cleaved fragments (MsPO1, 72.86 kD; MsPO2, 74.14 kD) have very low PO activity. When SPHs were added, PO activity increased significantly (Yu et al., 2003) (Figure 1B). *M. sexta* SPH-1 and SPH-2 associate loosely with PAP1 (or PAP3) and PPO to form a large complex (Wang and Jiang, 2004; Gupta et al., 2005). Based on the crystal structure of MsPPO2 and the computer-modeled structures of activated MsPO2, the surface electrostatic charge is changed from largely negative to mainly positive when the N-terminal sequence is cleaved at R^51^ (Li et al., 2009). PAP-2 has three positively charged areas in its dual-clip domains (Huang et al., 2007), one of which may first associate with MsPPO in the negatively charged region. When the N-terminal sequence is cleaved by PAP, the exposed positively charged region is ready for the SPH association since, according to a study in *H. diomphalia* (Piao et al., 2005), SPH has a negatively charged surface in the clip domain. The association of SPHs with activated MsPO may induce MsPO domain I into conformational changes, dislodging the place holder from the active site pocket and allowing substrates to enter (Li et al., 2009). In *M. sexta*, hemolymph proteinases HP14 and HP21 are involved in the MsPPO activation cascade (Ji et al., 2004; Wang and Jiang, 2006; Gorman et al., 2007). Active HP14 cleaves ProHP21 to produce active HP21, which then cleaves ProPAPs to produce active PAPs (Kanost et al., 2004; Kanost and Gorman, 2008). Serpin-3, serpin-4, serpin-6, and serpin-1J negatively regulate each step of the cascade (Kanost et al., 2004; Kanost and Gorman, 2008).

### Mechanism 3

The PPO activation pathway of *H. diomphalia* has also been determined. In *H. diomphalia*, three PPO-activating factor (PPAF-I, PPAF-II, and PPAF-III) have been identified (Lee et al., 1998; Kim et al., 2002). PPAF-II is similar to *M. sexta* SPH and contains a non-catalytic clip domain (Lee et al., 1998; Kim et al., 2002). As with *B. mori* PPAE and *M. sexta* PAPs, PPAF-I cleaves HdPPO-I at the conserved Arg-Phe (^50^RF^51^) bond, and the large fragment (76 kD) is inactive (Lee et al., 1998; Kim et al., 2002). However, when all PPAFs were mixed with purified HdPPO-I in a buffer containing Ca^2+^, a new fragment (60 kD) with PO activity was produced in addition to the 76-kD fragment (Lee et al., 1998; Kim et al., 2002). N-terminal sequencing shows that the 60-kD fragment was cleaved between ^162^RA^163^ in HdproPO-I. Therefore, in *H. diomphalia*, HdPPO is not activated until it is cleaved at two regions (Figure 1C). Based on the MsPPO crystal structure, the second cleavage occurs after the place holder, and exposes the active site pocket (Li et al., 2009).

α-chymotrypsin is a typical serine protease and, when used to cleave BmPPO, resulted in PO activity (Ohnishi et al., 1970). *Galleria mellonella* PPO (GmPPO) was also mixed with α-chymotrypsin but no PO activity was even indicated (Kopácek et al., 1995). Another study mixed purified recombinant DmPPO1 with trypsin and α-chymotrypsin separately, and found that α-chymotrypsin cleaved DmPPO1 into two bands after separation on a native gel: one (~60 kD) with direct PO activity, and the other (~76 kD) that requires activation by ethanol (Lu et al., 2014a). When His-tag was added to DmPPO1 at either the N- or C-terminus and evaluated using antibodies against His-tag, no signals were detected with the two bands. This indicates that α-chymotrypsin initially cleaves DmPPO1 at both the N- and C-terminus simultaneously. The 76-kD band requiring ethanol activation was produced rapidly, with a mass ratio of DmPPO1 and α-chymotrypsin at 70:1, and DmPPO1 disappeared within the assayed time. Next, the mass ratio of DmPPO1 to α-chymotrypsin was changed to 1:1, and the 76-kD band was further cleaved after the place holder to produce a 60-kD fragment with direct PO activity. Thus, α-chymotrypsin cleaved DmPPO1 in at least three places, producing active DmPO1(60 kD): first at the N and C-termini simultaneously to produce DmPPO1(76 kD), and second after the place holder to form DmPO1(60 kD) (Lu et al., 2014a), as summarized in Figure 1D. In this work, the 3D view of different potential cleaving sites were also compared in a supplementary picture (Lu et al., 2014a), which may be helpful to understand the cleavage and activation. In *Aedes aegypti*, two immune melanization proteases (IMP-1 and IMP-2) induce melanization against parasites, cleaving Arg or Lys around the 162-position and producing active AaPO (50 kD) (Zou et al., 2010). In *A. aegypti*, a purified fragment with a molecular weight of 60 kD had PO activity upon activation using detergents or 2-propanol. According to a LC-MS/MS assay, this fragment contained PPO1, PPO2, and PPO3 fragments (Li et al., 2005). This might be due to mRNA splicing among the three PPOs. Activation of DmPPO1 by α-chymotrypsin indicates that it is possible for PPO to be directly cleaved for activation. However, further work will be necessary to determine whether there is a serine protease that functions similar commercial α-chymotrypsin in insects, as the described work was performed *in vitro* (Lu et al., 2014a).

**Figure 2:**
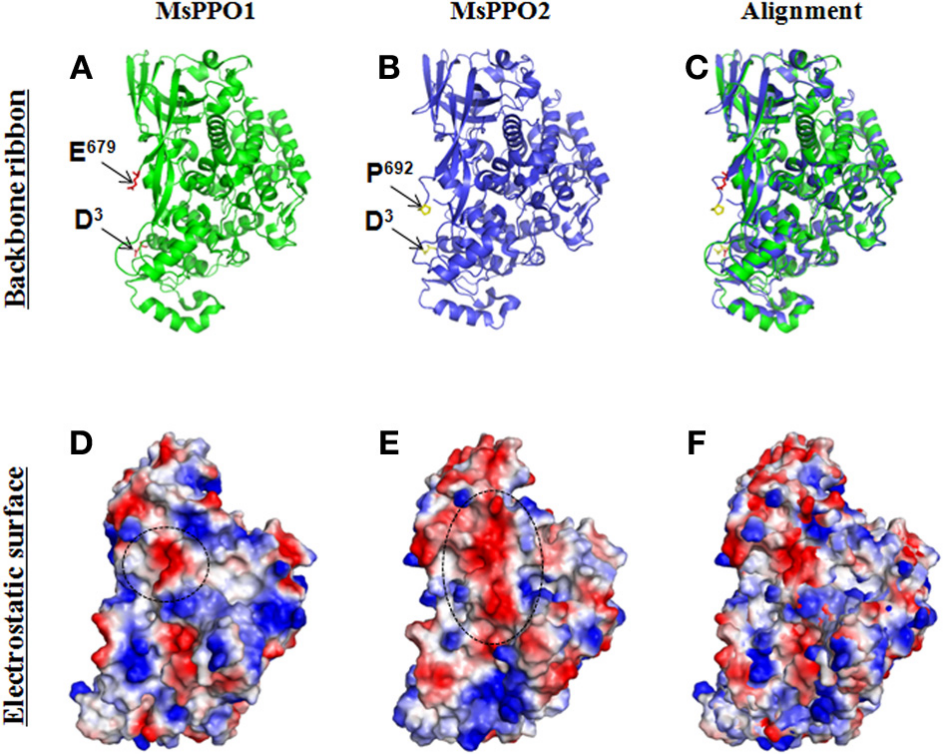
**Electrostatic surfaces of MsPPO1 and MsPPO2**. Crystal structures of two *Manduca sexta* PPOs (PDB ID code 3HHS) were aligned using the PyMOL Molecular Graphics System (http://pymol.org/). **(A–C)** Secondary structure (Backbone ribbons) of two *M. sexta* PPOs. PPO1 crystal structure **(A)**. The N-terminus residue is D^3^, and C-terminus residue is E^679^ as indicated by arrows. PPO2 crystal structure **(B)**. The N-terminus residue is D^3^, and C-terminus residue is E^692^ as indicated by arrows. **(C)**. Alignment of PPO1 and PPO2 at the same angle. **(D,E)** The electrostatic surfaces of two *M. sexta* PPOs after being generated using the PyMOL software. The backbone ribbons and electrostatic surfaces of each PPO are surveyed from the same view. Red is negative, and blue is positive. MsPPO1 **(D)** and MsPPO2 **(E)** have different surface electrical charges. In **(D)**, the circled negative area is composed of I^97^D^98^, A^221^D^222^ residues. In **(E)**, the circled negative area is composed of N^97^E^98^, D^101^, S^225^A^226^S^227^, E^229^, V^232^, S^355^V^356^L^357^ residues. **(F)**. Alignment of PPO1 and PPO2 at the same angle.

Trypsin is another typical serine protease. According to predictions using the GPMAW software (http://www.gpmaw.com/), trypsin can precisely cleave DmPPO1 at ^52^RF^53^ and ^16^RD^165^ (Lu et al., 2014a), which correspond to ^51^RF^52^ in BmPPO1 and ^162^RA^163^ in HdPPO-I (Ashida and Brey, 1997; Lee et al., 1998; Kim et al., 2002). Therefore, in prediction, trypsin should cleave DmPPO1 to produce DmPO1 with a molecular weight of either 76 kD (as in *B. mori* and *M. sexta*) or 60 kD (as in *H. diomphalia*). Results showed that trypsin cleaved DmPPO1 into a 60-kD fragment that could be further activated by ethanol but not by α-chymotrypsin (Lu et al., 2014a). Therefore, the protein structure of insect PPO likely affects its activation by serine protease cleavage. Based on their crystal structures, MsPPO1 and MsPPO2 differ in their surface electric charge and structure (Figure 2), which may complicate PPO activation since the electrostatic interactions of MsPPO, SPH, and PAPs are involved in the activation process (Li et al., 2009). In addition to serine protease, detergents such as sodium dodecyl sulfate (SDS), CPC, and ethanol can activate insect PPO (Asada et al., 1993; Ashida and Brey, 1997). Cationic detergents are thought to interact with PPO through electrostatic interaction, causing conformational changes and dislodging the place holder (Li et al., 2009). Since it is easy to obtain large amounts of recombinant PPO by expression in *E. coli*, the mechanism of ethanol activation will likely be elucidated by means of determination of crystal structures.

DmPPO3 is a unique PPO in *D. melanogaster*, since it displays the direct PO activity that cannot be explained by the mechanisms described above (Nam et al., 2008; Liu et al., 2012). PPO released into the hindgut of *B. mori* directly induces feces melanization (Shao et al., 2012), but it is unclear how BmPPO is activated in the hindgut. Some proteinases have been identified in larval hindgut contents (Shao et al., 2012); these may be involved in PPO activation in the hindgut.

In a word, PPO activation in insects is much more complicated than we understood. Many more serine proteases are probably involved in PPO cleavage and activations in one of mechanisms as described above. With the genome of many insects available, it is the time to do a screening to see how many serine proteases or even other proteases that can cleave insect PPO for activation.

## Properties of active PO and its negative regulation

α-chymotrypsin cleaves recombinant DmPPO1 into a fragment known as DmPO1(60 kD), which has direct PO activity (Lu et al., 2014a). Apo-DmPPO1 can be purified without the addition of Cu^2+^ in the culture medium. Active PO is easily adsorbed on the surfaces of many materials and forms aggregates with itself and other molecules (Ashida and Brey, 1997). When used for PPO activation, apo-DmPPO1 can overcome the associated properties of PO, resulting in the production of active DmPO1(60 kD) upon addition of Cu^2+^. DmPO1(60 kD) was lost on the native gel after incubation with dopamine, likely because it formed large molecules with dopamine, as suggested by SDS-PAGE (Lu et al., 2014a). DmPO1(60 kD) activity was not affected by high temperature (37°C) or NaCl (high concentrations), and staining of a native gel showed that EDTA did not chelate Cu^2+^ in the active site pocket. Notably, DmPO1(60 kD) separated on a native gel associates into a ~260 kD molecule upon resolution by denaturing SDS-PAGE (Lu et al., 2014a). In *B. mori*, the wound activated PO oxidizes Tyr residues in other proteins to produce o-quinones that can be covalently cross-linked with PO and several other proteins to form large complex (~670 kD) (Clark and Strand, 2013). This complex can use endogenous Tyr for rapid melanization.

During the melanization process, many toxic molecules—such as cytotoxic quinones and reactive oxygen—are produced (Christensen et al., 2005; Nappi and Christensen, 2005). In most cases, melanization is limited to invading microorganisms. Uncontrolled and systematic melanization is also lethal to insects and, therefore, insects have many means of regulating melanization. Serpins regulate the time and locations of melanization by inhibiting the corresponding protease activity, which occurs before the PO formation (Kanost and Gorman, 2008). Additionally, several small peptides or large proteins have been shown to regulate PO activity, as does a 4-kD hemolymph peptide from *Musca domestica, Anophele gambiae*, and *M. sexta* (Tsukamoto et al., 1992; Sugumaran and Nellaiappan, 2000; Lu et al., 2008). A 380-kD PO inhibitor from *M. sexta* larval cuticles, and a 43-kD melanization inhibitor from *Tenebrio melitor*, also inhibit PO activity, thus avoiding systematic melanization in the hemolymph (Sugumaran and Nellaiappan, 2000; Zhao et al., 2005). However, the exact mechanisms by which these proteins inhibit PO activity are unclear.

In *A. aegypti*, CLSP2 containing C-type lectin (CTL) and elastase-like SP domains reduced hemolymph melanization. When *A. aegypti* adults underwent acute infection, CLSP2 reduced hemolymph melanization (Shin et al., 2011). In CLSP2, the CTL domain is thought to function in pathogen detection, and the ESP domain is likely involved in regulating melanization. *Plasmodium gallinaceum* infection up-regulated CLSP2 gene transcription, and an RNAi assay showed that CLSP2 was required for parasite development. Thus, CLSP2 reduces PPO activation in response to parasite infection, which is advantageous to parasite development.

Lysozyme can also regulate PO-induced melanization. In *A. gambiae*, the binding of lysozyme C-1 inhibited Sephadex bead melanization (Li and Paskewitz, 2006). *M. sexta* lysozyme interacts with plasma PPO directly (Rao et al., 2010). Incubating naïve plasma with lysozyme inhibited the conversion of MsPPO to PO. However, PAP1 and SPH2, two important components of the MsPPO activation pathway, were degraded. This trade-off between lysozyme and PO activity was also observed in *Spodoptera littoralis* larvae (Cotter et al., 2008), wherein dark lines had higher lysozyme activity but PO activity was decreased, and pale lines had lower lysozyme but higher PO activity. Therefore, lysozyme is another negative regulator of PPO activation, which probably functions via interaction with PPO.

Several proteins and small molecules produced by insect pathogens have been found to regulate PO activity and melanization. *Microplitis demoliter* bracovirus (MdBV) produces a protein known as Egf 1.0 that has the same cleavage sequence, Arg-Phe, as insect PPO for activation (Ashida and Brey, 1997; Beck and Strand, 2007). Egf 1.0 binds to *M. sexta* PAPs through its C-terminal repeat domain, and it can prevent pro-PAP and pro-SPH from activating PPO (Beck and Strand, 2007; Lu et al., 2008). The insect pathogen *Metarhizium robertsii* produces 39 cyclohexadepsi-peptide destruxins (dtxs) that can suppress both the cellular and humoral immune responses, especially hemolymph melanization (Wang et al., 2012). When either Dtx S1 or Dtx S2 was deleted from *M. robertsii* that could not produce dtxs, hemolymph from the infected insects could become melanized as naïve larvae in the air. This indicates that dtxs inhibits hemolymph PPO activation. *Photorhabdus luminescens* is a Gram-negative bacterium, and produces a small molecular antibiotic, (E)-1,3-dihydroxy-2-(isopropyl)-5-(2-phenylethenyl)benzene (ST), that can also inhibit *M. sexta* PO activity (Eleftherianos et al., 2007). PO inhibitors produced by insects help to avoid harmful systematic melanization. However, PO inhibitors produced by pathogens allow them to escape the immune responses of host insects.

## PPO functions beyond immunity

Insect PPO is an important innate immunity protein protecting hosts from infection, since it is rapidly activated *in vivo* (Hillyer et al., 2003, 2004). Insect PPO is also important for wound healing (Binggeli et al., 2014). However, many studies have shown that insect PPO has some functions beyond immunity. A recent study shows that epidermal cells in the *B. mori* hindgut can produce BmPPO and release it into the hindgut contents (Shao et al., 2012). Hindgut BmPPO is the reason that phytophagous insects excrete black feces despite consuming green leaves. When PO activity in the hindgut was inhibited by phenylthiourea (PTU), insects excreted green feces. Furthermore, these green feces contained many more bacteria than did black feces. Therefore, insects use the hindgut PPO to exterminate potential pathogens in the feces through melanization, thereby protecting their food from pollution by feces transferred pathogens. Another recent study shows that melanization triggered by active Hayan in *D. melanogaster* can activate the c-Jun-N terminal kinase (JNK) in neuronal tissues (Nam et al., 2012). When the integuments of adult *D. melanogaster* were experimentally damaged, inactive Hayan was activated and hemolymph PPO was cleaved to produce PO. PO-induced melanization yielded reactive oxygen species (ROS) that activate the neuronal JNK pathway and protect against further injury. Thus, the hemolymph Hayan-PO cascade links wound and neuron responses via ROS produced by melanization (Nam et al., 2012). In crayfish, when proPO was cleaved by proPO-activating enzyme (ppA), the N-terminal portion separated from PO displayed antibacterial activity, accompanying the agglutination of bacteria and altering cell morphology (Jearaphunt et al., 2014). Furthermore, crayfish proPO can also be cleaved by caspase-1L to produce two N-terminal portions, proPOcasp1 (43 kD) and proPOcasp2 (45 kD), that also exhibit antibacterial activity. This work links caspase-induced inflammation and melanization for the first time. Melanization induced by PO activation combats microbial infections. In *D. melanogaster*, CG3066 is an indirect protease involved in activation of PPO to induce melanization. CG3066 mutants that lack PO activity had variable resistance and tolerance to various pathogens (Ayres and Schneider, 2008), indicating that their immune responses were tuned by evolution. These results indicate that many biological phenomena in insects are closely related to PPO and/or melanization. However, the focus of research should not be an immunity alone in the future.

Insect PPO and PO-induced melanization may affect insect longevity. *D. melanogaster* mutants with DmPPO1 and/or DmPPO2 deleted display novel phenotypes (Binggeli et al., 2014). Double mutants (DmPPO1^Δ^, DmPPO2^Δ^) had significantly shorter longevity (half-life = 24 days) than did the wild type, or DmPPO1^Δ^ and DmPPO2^Δ^ single mutants. DmPPO1^Δ^ and DmPPO2^Δ^ single mutants were also shorter-lived than wild-type *D. melanogaster*. Identical results were observed under germ-free conditions. In *S. littoralis*, PO activity affects larval development time (Cotter et al., 2008). Pale lines have higher PO activity and take longer to develop into adults, while dark lines have lower PO activity but develop more rapidly. Yellow dung flies, *Scathophaga stercoraria*, were successfully selected to obtain separate lines with low and high PO activity (Schwarzenbach and Ward, 2006). Flies with high PO activity lived slightly longer than both the control and those with low PO activity. However, flies with high PO activity died earlier than others when starved. In *A. gambiae*, adult longevity was reduced when serpin-2 (SRPN-2), but not its interactions, CLIPB9, was knocked down (An et al., 2011). SRPN-2 is a key component in negatively regulating melanization, and knock down of SRPN-2 induced melanotic pseudotumors in adults. Melanization induces the production of cytotoxic semiquinones and reactive oxygen intermediates, which likely contribute to reducing longevity (An et al., 2011). However, double knock-down of SRPN-2 and CLIPB9 rescues the phenotype induced by SRPN2 silencing (An et al., 2011). The interaction of SRPN-2 and CLIPB9 clearly influences adult longevity. Thus, insect PPO, including melanization induced by PO, affects insect longevity.

The PPO may also affect insect development. *B. mori* larval wing discs and *T. castaneum* adult hind wings contain PPO proteins with unknown function (Diao et al., 2012; Dittmer et al., 2012). However, when prophenoloxidase III was knocked down in *Armigeres subalbatus*, the adult wings became abnormal (Tsao et al., 2009). Additionally, knock-down of *A. subalbatus* pro-PO III caused the incomplete formation of pupal endocuticles and pharate adult cuticles (Tsao et al., 2010), indicating that pro-PO III is a prerequisite for adult mosquito development. Furthermore, yellow dung flies with high PO activity had larger clutches and laid many more eggs than control insects, or those with low PO activity (Schwarzenbach and Ward, 2006). PPO was detected in *B. mori* silk glands using liquid chromatography-tandem mass spectrometry (Dong et al., 2013); PPO and other proteins, such as laccase and peroxidase, appeared to cross-link the extracellular matrix. The tanning of the chorion of newly laid mosquito eggs occurs rapidly to avoid collapse, most likely due to evaporation under high summer temperatures. PPO was also detected in the mosquito eggshell (Li and Christensen, 1993; Marinotti et al., 2014). Chorion tanning is initiated by activating PO catalyzing L-tyrosine into L-DOPA (Li and Christensen, 1993), after which L-DOPA decarboxylase (DDC) decarboxylates L-DOPA into dopamine. L-DOPA and dopamine are oxidized by PO, tanning the egg chorion. When PO activity was inhibited, egg tanning was delayed. Therefore, PPO is also closely related to insect development.

Generally, when insects are infected by pathogens, their plasma PO activity will change (González-Santoyo and Córdoba-Aguilar, 2012). DNA microarray analysis of *A. gambiae* shows that AgPPO5, AgPPO6, and AgPPO9 are expressed rhythmically (Rund et al., 2011). Other proteins, such as several C-type lectins, clip-domain serine protease, and PGRP—that are involved in melanization are also expressed rhythmically, indicating that parasites in mosquito adults may adapt to the daily rhythm of host immunity (Rund et al., 2011). In *A. aegypti*, the expression of four PPO genes (AaPPO1, AaPPO3, AaPPO5, and AaPPO8) was activated by RUNT-related transcription factor 4 (RUNX4) upon microbial infection, under regulation by the Toll pathway (Zou et al., 2008). RUNX4 may also act cooperatively with REL1 to fight avian *P. gallinaceum* infection in adults.

There are up to 10 PPO genes in mosquitoes (Christophides et al., 2002; Waterhouse et al., 2007). It is unknown whether each PPO gene in mosquitoes and other insects has a novel function, and DmPPO was investigated with this in mind. *In vitro* (Asano and Takebuchi, 2009; Chen et al., 2012; Liu et al., 2012) and *in vivo* (Binggeli et al., 2014) studies have clarified many properties of each PPO in *D. melanogaster*. All three PPOs, including their corresponding PPO-GFP fused proteins, are expressed in *E. coli* or S2 cells with or without Cu^2+^ addition (Yang et al., 2013). DmPPO3 is unique since it can be auto-activated due to the presence of key amino acids and the loss of one fragment (Chen et al., 2012). DmPPO1 generally has higher PO activity than DmPPO2 when activated by ethanol or AMM1 (Li et al., 2012; Liu et al., 2012). DmPPO3 has the highest capacity for Cu^2+^ association, while that of DmPPO1 is intermediate and DmPPO2 associates with Cu^2+^ only at high concentrations. Separate deletions of DmPPO1 and DmPPO2 from *D. melanogaster* show that DmPPO1 exists mainly in the hemolymph, while DmPPO2 occurs within the crystal of crystal cells (Binggeli et al., 2014). DmPPO1 has antibacterial activity *in vitro* (Lu et al., 2014b). DmPPO1- and DmPPO2-deletion mutants show that DmPPO1 is more important than DmPPO2 in wound healing and defense against Gram-positive bacteria and fungi (Binggeli et al., 2014). Obviously, each insect PPO may have different properties. However, we neglect the study due to our attention on PPO activation change.

## Molecular evolution of insect PPO

**Figure 3:**
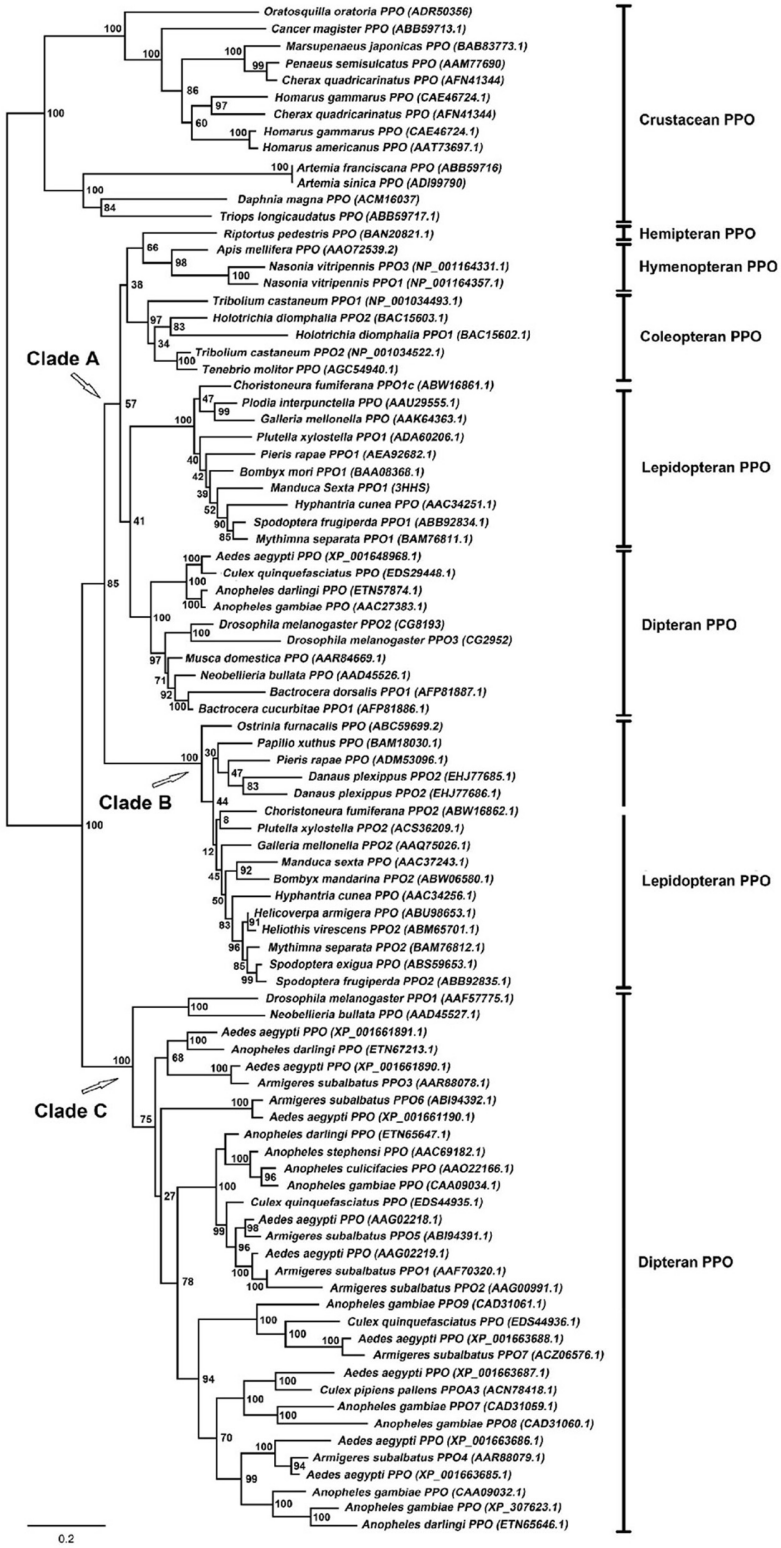
**Maximum-likelihood tree of insect prophenoloxidases calculated using the RAxML method via the CIPRES Science Gateway (Miller et al., 2010)**. Crustacean PPOs were used as an outgroup. The PPO genes from various insect species are indicated along with their NCBI accession numbers.

Insects are abundant animals, and each species of insect possesses at least one PPO gene. Mosquitos, for example, possess up to 10 PPO genes in the genome (Christophides et al., 2002; Waterhouse et al., 2007). Using “prophenoloxidase” as a keyword in a search of the National Center for Biotechnology Information (NCBI; http://www.ncbi.nlm.nih.gov/) database resulted in recovery of at least 75 insect PPO genes with full sequences deposited to date. A phylogenic tree for those insect PPOs was analyzed. The randomized axelerated maximum-likelihood (RAxML) method (The Exelixis Lab, Heidelberg, Germany) was used to reconstruct the phylogenetic relationships of insect PPOs based on amino acid sequences via the Cyberinfrasctructure for Phylogenetic Research (CIPRES) Science Gateway, with crustacean PPOs as the outgroup (Miller et al., 2010) (Figure 3). The phylogenetic structure indicates three major clades of insect PPO: Clade A comprises the conserved PPOs distributed among various insect orders, whereas Clades B and C represent distinctive paralogs specifically occurring only in Lepidoptera and Diptera, respectively. In one unusual case, the Hemiptera PPO *Choristoneura fumiferana* PPO2 (ABW16862.1) was assigned to Clade B. The origin and timing of the evolution of three separate PPO clades is unknown, and requires further study.

Arthropod PPO, hemocyanin, hexamerin, pseudohemocyanin, and hexamerin receptor amino acid sequences are similar (Burmester, 2002). These proteins serve different physiological functions, but all belong to the hemocyanin superfamily (Burmester, 2002) and contain ~700 amino acids each (Burmester, 2002). Insect PPO is an important innate immunity protein that is involved in cellular and humoral immune responses (Kanost et al., 2004; Cerenius et al., 2008; Kanost and Gorman, 2008). Following activation, PPO induces melanization around invading pathogens and wounds (Kanost et al., 2004; Cerenius et al., 2008; Kanost and Gorman, 2008). Hemocyanins are found in some arthropods and mollusks, and serve to transfer oxygen to tissues (van Holde and Miller, 1995; Kusche et al., 2002). The amino acid sequences of insect PPO and hemocyanin are highly conserved (Burmester, 2002). Each hemocyanin has two copper-binding sites, and each copper ion is coordinated by three histidines. Two copper ions in hemocyanin are a prerequisite for binding with oxygen (van Holde and Miller, 1995). Colorless hemocyanin turns blue after binding to oxygen (van Holde and Miller, 1995). Hemocyanin demonstrates PPO activity upon treatment with a detergent such as SDS (Decker et al., 2001). Hexamerin is widely accepted as a type of storage protein that primarily supplies nutrients during metamorphosis (Burmester and Scheller, 1999). Hexamerin is derived from hemocyanin (Burmester, 2002). Although it has no copper-binding sites, its amino acid sequence and protein structure are similar to those of PPO and hemocyanin (Willott et al., 1989). Hexamerin of the Coleoptera, Diptera, and Lepidoptera contains abundant aromatic amino acids, and is also known as arylphorin in these insect orders (Burmester and Scheller, 1999). Some Lepidoptera hexamerin also contains high amounts of methionine (Burmester, 2002).

**Figure 4:**
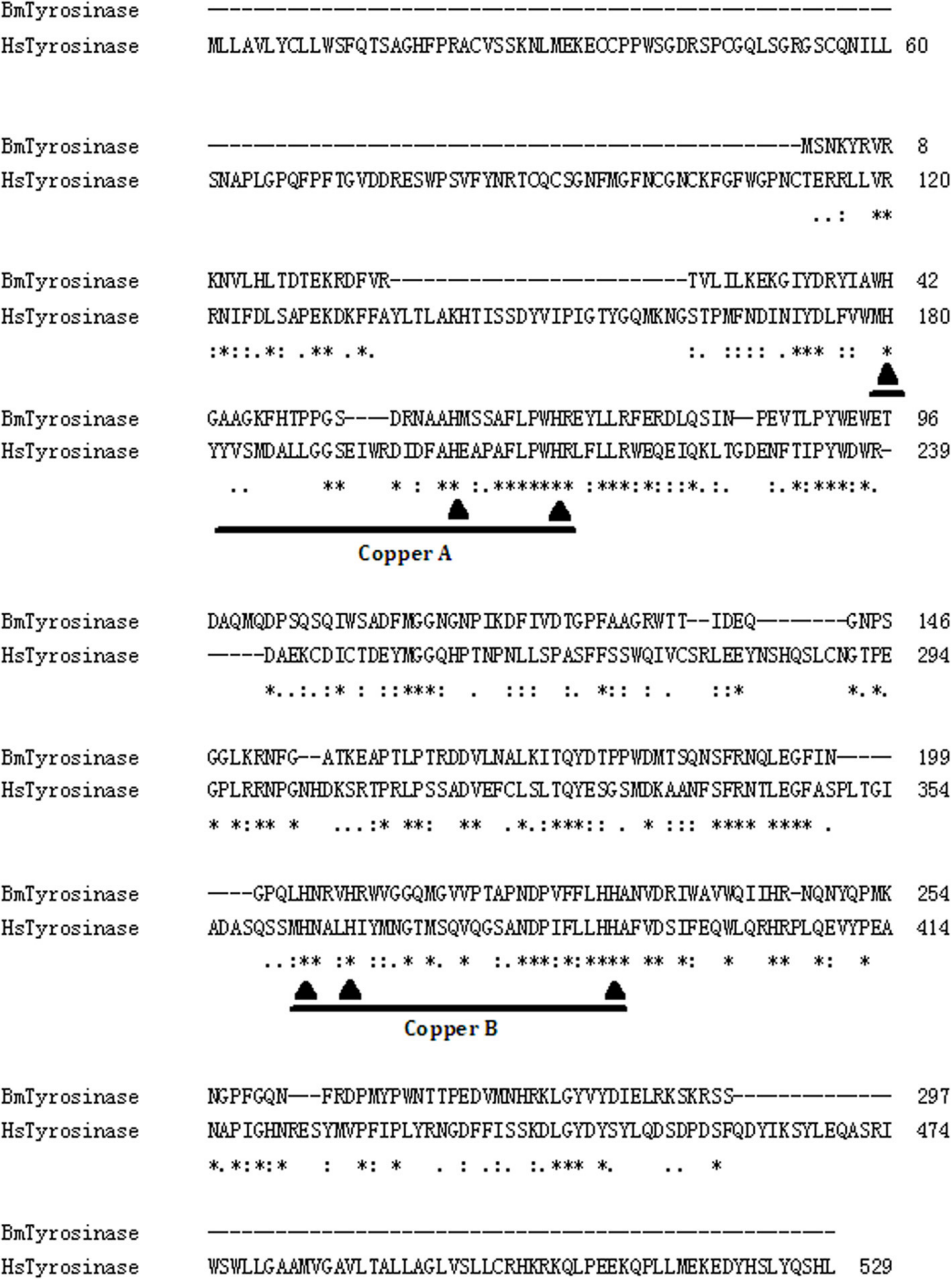
**Comparison of the amino acid sequences of *Homo sapiens* tyrosinase (HsTyrosinase; Accession number: AAB60319) and *Bacillus megaterim* tyrosinase (BmTyrosinase; Accession number: ACC86108)**. The online CLUSTALW multiple sequence alignment software (http://www.genome.jp/tools/clustalw/) was used for the slow and accurate pairwise alignment analysis. “^*^,” Fully conserved residues; “:,” conserved substitutions; “.,” semi-conserved substitution as previously described (Grasela et al., 2008). Copper-binding regions A and B are underlined. The histidine residues marked with “▲” are the ligands proposed to coordinate two copper ions in the active site pocket.

The evolution of the type-3 copper protein has been analyzed elsewhere (Aguilera et al., 2013; Singh et al., 2013). Various types-3 copper proteins have certain similarities among amino acid sequences. Type-3 copper proteins are classified into α- (secreted form), β- (cytosolic form), and γ-subclasses (membrane-bound form) (Aguilera et al., 2013). Insect PPO belongs to β- subclasses. The α-subclass appeared early, while the other two appeared later through gene duplication. So it is still unclear when and why insect PPO evolved to function as an immunity protein. Very interestingly, we also found that the amino acid sequences of *Homo sapiens* tyrosinase (Accession: AAB60319) and bacterial (*Bacillus megaterium*) tyrosinase (Accession: AAB60319) even exhibit around 30% similarity (Figure 4). It is uncertain whether the tyrosinase of microorganisms and the tyrosinase of advanced animals share an evolutionary relationship, or if its occurrence in both groups is coincidental. As each type-3 copper protein functions differently in different organisms, it is important to understand when and why these proteins evolved to fulfill their various physiological functions.

## Future prospects

In *D. melanogaster*, each PPO has unique properties and even functions. But the question remains: why do mosquitoes need so many PPO genes? Certainly, PPO and the melanization induced by activating PO are important factors in fighting malaria *in vivo* (Collins et al., 1986). However, it is unclear if they have novel functions such as those described for *A. subalbatus* prophenoloxidase III (Tsao et al., 2009, 2010). It is likely that the various insect PPOs serve as-yet-unknown functions. As a typical copper 3 protein, insect PPO has been investigated for a long time (Ashida and Brey, 1997). Through outstanding research on PPO in *B. mori, M. sexta, H. diomphalia, D. melanogaster*, mosquitoes, and other invertebrates, it is clear that insect PPO is a critical factor in the defense against invading pathogens due to its rapid activation (Hillyer et al., 2003, 2004; Cerenius et al., 2008; Kanost and Gorman, 2008). Much is now understood about the PPO activation cascade in hemolymph (Kanost and Gorman, 2008). *D. melanogaster* has been used for investigation of the PPO activation pathway, and many important proteins that regulate PPO activation have been identified. The MP1, MP2, Hayan, and CG9737 proteases can cleave DmPPO (Tang, 2009; Nam et al., 2012; An et al., 2013). *D. melanogaster* serpins, such as Spn27A and Spn28D, negatively regulate PPO activation and melanization (Christensen et al., 2005; Nappi et al., 2005; Scherfer et al., 2008), while MP2 and Spn27A act together to regulate PPO activation (An et al., 2013). However, much about *D. melanogaster* PPO activation remains unclear. Some researchers will likely take advantage of *D. melanogaster* genetics and biochemistry to clarify the PPO activation pathway in *D. melanogaster*.

In *B. mori*, RNAi and recombinant Reeler injection recently showed that an immunity-induced Reeler is involved in the PPO activation pathway (Bao et al., 2008). Other novel proteins may be also involved in insect PPO activation, but this requires more research. In *M. sexta*, 163 conserved and 13 novel microRNAs have been identified, some of which may regulate the expression of genes in the PPO activation pathway (Zhang et al., 2014). Thus, the influence of microRNAs on insect PPO activation is an interesting subject for future research. Immune capacity and even longevity were seriously affected by knock-down and knock-out (deletion) of insect PPO (Binggeli et al., 2014), which indicates that insect PPO is an important factors for immunity protection. In contrast, pathogens secrete various small molecules to inhibit PO activity (Eleftherianos et al., 2007; Wang et al., 2012), which is advantageous for pathogens to escape host immunity. In future, it may be possible to apply these principles to pest control or production of beneficial insects and other invertebrates.

### Conflict of interest statement

The authors declare that the research was conducted in the absence of any commercial or financial relationships that could be construed as a potential conflict of interest.
